# Diseases of Nose and Throat

**Published:** 1896-04

**Authors:** Henry J. Mulford

**Affiliations:** Clinical instructor in diseases of the nose and throat, Medical Department, University of Buffalo


### Progress in Medical Science.

### DISEASES OF NOSE AND THROAT.

Reported by HENRY J. MULFORD, M. D.,

Clinical instructor in diseases of the nose and throat, Medical Department, University of Buffalo.

### ADENOID VEGETATIONS.

IN A PAPER, read last year before the Fifth International Congress of Otology at Florence, Dr. Y. Arslan, of Padua, states his conclusions as to etiology and treatment of "adenoids," after having seen over four hundred cases. Amongst 4,080 patients suffering from affections of the nose, throat or ear, 426 had adenoid tumors in the vault. Of these 69 per cent. had symptoms of nasal obstruction, 37 per cent. suffered with tonsilitis or pharyngitis, 59 per cent. had ear complications, of whom 110 were cases of suppurative otitis and 142 were cases of deafness without suppuration. Of six deaf mutes with adenoids two were benefited by their removal. Other complications noted were bronchitis, laryngeal spasm, night terrors, stammering, nocturnal enuresis and convulsive attacks. A case of Jacksonian epilepsy, thought to be of central origin, soon disappeared after operation.

Much importance is attributed to heredity and general diseases as causes. In sixty cases, noted traces of heredity. Dampness and other causes of secondary importance.

Treatment consists in complete removal. Advisable to operate even when the masses are small. The operation should be completed at one sitting. As regards direct complications, the operation is certain in its benefits ; for reflex complications the result is not so positive. Disappearance does not always take place with age, for the growths were found in patients ranging from twenty to forty years.

General narcosis should be employed, otherwise a simple operation is made complicated, long and brutal, especially as the majority of our patients are children, in whom we cannot expect complete docility. Cocaine insufficient. Of the anesthetics gives preference to ethyl bromide. Superior to chloroform or ether for short operations. It is rapid and certain in its action, harmless in the dose employed (10 to 20 grammes), and leaves no disagreeable consequences. Gas might do as well, but not so handy. Collected 4,161 cases of anesthesia by ethyl bromide, in which some unsuccessful cases were noted, but not one death. Has used it 252 times without seeing the least disagreeable symptom. It should be given by what is called the intensive method. Up to fifteen years, 10 grammes are enough ; above that age the dose may be doubled.

Prognosis good. Nasal obstruction disappears. The healing of suppurating otitis, unless there is extensive caries, is hastened. In deafness without suppuration, nearly always rapid recovery of hearing. In adults, secondary lesions of tympanum and ossicles may be unchanged. In deaf mutes, when young, good results may follow. Often in reflex complications unexpected and brilliant results may be obtained.

*Conclusions.*—(1) In Italy this condition is fairly frequent. (2) The chief causes are heredity and general affections. (3) The operation should be completed in one sitting. (4) Ethyl bromide the best anesthetic. (5) Auricular affections are in great part due to adenoid vegetations, both during the period of development of the latter and during their retrogression. (6) In all cases where adenoid tumors have been diagnosticated there should be no delay in their removal. (7) Before children are admitted into asylums for the deaf and dumb, or similar institutions, they should first be submitted to examination by a specialist.—(Abstract of translation published in the *Journal of Laryngology, Rhinology and Otology* for December, 1895.)

---

THE CIGARETTE HABIT.

Abstract of paper by Dr. Mulhall, with discussion, read at last meeting of American Laryngological Association. From the *Journal of Laryngology, Rhinology and Otology :*

Chewers, smokers and snuff-takers each derive a special satisfaction from the use of tobacco. Cigarette smokers, from habit of inhaling, derive more pleasure than cigar or pipe smokers. The smoke does not penetrate into pulmonary structure beyond the first division of the bronchi. The smoker used to certain degrees of satisfaction does not find it in either a milder or stronger cigarette or in a cigar. The feeling experienced is a pleasurable irritation of the laryngeal and tracheal fibers of pneumogastric nerve. It is a nicotine satisfaction.

Amount of nicotine absorption varies according to the extent of surface, which in inhalers is three times that of noninhalers. Three cigarettes have the nicotine strength of one cigar, and there is no reliable evidence to prove that cigarettes are adulterated with opium or other deleterious drugs. Cigarette smoking is a "deadly" habit, because of its frequency. Its effects are analogous to those of giving a drug in small and frequent doses.

The constitutional effects are those from tobacco in any form, always nicotinism. On the young the results are most pernicious. Locally it may aggravate preëxisting trouble, but it rarely originates any disease. There may result a slight hyperemia of the mucosa, or a slight catarrh, with pearly secretion ejected in small pellets with a single slight cough. Once in a while a whistling râle is heard over the bronchi, but only in the case of deep and excessive inhalers.

Mario, the great tenor, inhaled constantly and between the acts of the opera. Maxwell, the St. Louis murderer, while in prison inhaled forty cigarettes daily, and although he was a nervous wreck his throat did not show signs of disease, as was proven post mortem.

DISCUSSION.

Dr. Ingals could not accept the doctrine that tobacco did no harm to the throat, as he had seen pronounced tracheal cough in inhalers.

Dr. Seiler thought the habit of continual spitting was the real cause of the local trouble, as this led to abnormal dryness of the pharynx.

Dr. Langmaid believed that he could tell by the color of the mucosa of pharynx if a man smoked or not. Cigars have less effect on throat than pipes, owing to the heat in the stem of the latter and to the relatively larger mass of fire in bowl. Effect on the young of tobacco in any form, was especially destructive to power of consecutive thought. As to Mario, it was notorious that he never really exerted his vocal powers more than once a week. The rest of the time he intoned. As a general thing, tobacco is distinctly deleterious to finer qualities of the singing voice.

---

MICROÖRGANISMS IN THE HEALTHY NOSE.

Abstract of paper by Dr. St. Clair Thomson and Dr. Hewlett, of London, read before the Royal Medical and Chirurgical Society, London. Published in *Journal of Laryngology, Rhinology and Otology* :

Their results offer a striking contrast to those of the majority of previous observers, and directly oppose the opinion held by many physicians. About 500 liters of air, bearing on a low average 1,500 organisms, are inspired every hour. As the greater portion of this comes in contact with moist mucous membrane lining the nasal fossæ, it has been taken for granted that the interior of the nose must show a rich profusion of microörganisms.

The literature of the subject is gone over in chronological order. Only two papers found devoted entirely to bacteriology of normal nose ; other reference to the healthy state only made incidentally in the course of researches on diseased conditions. Only two authorities—Loewenberg and Hajek—find a scarcity of bacteria in the nose ; others record a greater or less variety and profusion. One observer finds the streptococcus of Fehlinsen present in one out of five healthy individuals ; another found the diplococcus pneumoniæ (Fränkel-Weichselbaum) one in four. The latter observer often met the bacillus pneumoniæ (Friedländer), the streptococcus pyogenes and the staphylococcus pyogenes aureus not only in large numbers but often in pure culture.

The method of examination adopted by the authors was cultivations on agar and cover-glass preparations stained with gentian violet. No attempt made to differentiate the organisms. This research dealt with the presence or absence of bacteria. Thirteen healthy individuals examined. Twenty-seven cultures and fourteen cover-glass preparations made from vestibule of nose. Seventy-six cultures and thirty cover-glass preparations made from mucous membrane of nasal cavity. Summary of results : (1) In all investigations of this kind a clear distinction must be made between the vestibule of the nose and the proper mucous cavity. The former lined with skin and furnished with hairs and sudoriferous and sebaceous glands; not a part of nasal cavity proper, but only leads to it. (2) Neglect of this distinction may account for the discrepancy in previous observations. (3) In the dust and crusts of mucus and debris found among the vibrissæ of healthy subjects, microörganisms are never absent. As a rule the number is abundant. (4) On the Schneiderian membrane the reverse is the case. It is not asserted that microörganisms are completely absent ; obviously some must occasionally occur, but normally they are never plentiful ; are rarely ever numerous. In more than eighty per cent. none whatever were found and the mucus was completely sterile. (5) The occurrence of pathogenic organisms must be so infrequent that their presence on the Schneiderian membrane can only be regarded as quite exceptional. Clinical experience bears out above conclusions, and their applications in practice is sufficiently obvious.

DISCUSSION.

Mr. SPENCER WATSON thought it premature to conclude that vestibule and vibrissæ filtered out nearly all the organisms in air entering nose. Since the vestibule was ill-developed and vibrissæ only represented by down in children, it was improbable that in them microörganisms were thus arrested. The authors might reply that children were in fact more subject to respiratory affections than adults. This might be so, but before drawing a definite conclusion as to this function of vestibule and its vibrissæ further observations on children were required. The greater part of the nasal fossæ was confessedly inaccessible to bacteriological investigation ; the anterior had been shown to be almost free from microörganisms, but it was possible that the microörganisms were carried past the smooth anterior parts, and came to rest on the more convoluted inaccessible parts. How was the automatic cleaning of the nose done ? Was it by filtration in the vestibule, or was it due to some active process in the deeper parts of the nasal fossæ, probably phagocytic in nature ? The rapidity and ease with which wounds of the nasal membrane healed, to some extent supported the author's views, but wounds of the face generally, and especially of the eyelids, healed readily, possibly a result of the free vascular supply of the parts.

Dr. F. SEMON agreed that wounds of nasal membrane healed rapidly, and did not think it necessary to use antiseptics before operating. It was remarkable how the nose escaped the inflammations common in the lower respiratory tract. Tuberculosis of the nose one of the rarest diseases, and suppuration rarely occurred after nasal operations. From a clinical standpoint he thought it unlikely that microörganisms passed over the parts accessible to examination and settled in the deeper parts.

Dr. ALLAN MACFADYEN : The authors had shown what an efficient filtering apparatus the nasal cavity was for impurities of all kinds. A good part of this result might be due to the mechanical action of the vibrissæ and the presence of sticky mucus. Still, a certain number of organisms must pass up, and the question arose as to what became of them. That aroused the inquiry as to the possible action of mucus upon organisms. Perhaps the mucus was not a suitable soil for their growth, or perhaps it had a bactericidal action. The investigations of Santorelli in respect to sputum had shown that most organisms lost their virulence under its influence, with the exception of the diphtheria baccillus.

Dr. HABERSOHN thought the paper had an especial bearing on the clinical and pathological side as to how far absorption might occur from healthy nasal membrane. A view was generally held that all portions of the mucous membrane covered with ciliated epithelium were not the first infected, this being explained on the ground that the cilia kept the organisms moving, and so prevented absorption. Some years ago he had studied this with regard to the larynx, and had found that the parts covered with ciliated epithelium were not so frequently affected.

Dr. HEWLETT, in reply, said that although many microbes were arrested by the vibrissæ, it was not stated that this was the essential factor which prevented their getting into the nose. With regard to self-cleansing, several factors were to be considered. The protective power of ciliated surface was an important factor ; another was the question of phagocytosis. Another point which some experiments seemed to bear out was that the nasal mucus was not a nutrient medium for microbes. This quality prevented their rapid multiplication, as occurred in the mouth. Then there was the mechanical action of the mucus which, always trickling over the surfaces, must carry down microbes and dust. With regard to the arrest of bacteria they had made many observations which had been rather a failure.

Dr. St. Clair Thomson, in reply, said the original purpose of their investigations had dealt with the character of the bacteria, but when it was found how rarely microörganisms of any kind were found in the nose, they decided to publish this fact by itself. The objection had occurred to him in regard to the vestibule of children's noses, and he had examined as many of their noses as possible. All of them were found to be lined with down, well moistened with mucus and often showed collections of dust. It was difficult to imagine that any microbes could escape impinging on the anterior mucous surfaces. With reference to the part played by ciliated epithelium he mentioned two observations by Sir Joseph Lister. The first referred to collections of blood and pus in the pleural cavity. When this communicated with the air through the bronchus the pleural contents became septic much less readily than in cases where there was communication with the air through chest wall. This difference he ascribed to the action of the ciliated epithelium. The second observation referred to cases of fracture of base of skull involving middle ear, with rupture of drum. Lister insisted on keeping the external meatus aseptic, as he held that most of the after-mischief was due to septic infection. When it was suggested that the entrance by way of the Eustachian tube was unprotected, he held that this approach was well guarded by its ciliated epithelium.

